# Climatic Effects on Planning Behavior

**DOI:** 10.1371/journal.pone.0126205

**Published:** 2015-05-19

**Authors:** Yong Liu, Vassilis Kostakos, Hongxiu Li

**Affiliations:** 1 Department of Information and Service Economy, Aalto University School of Business, Helsinki, Finland; 2 Community Imaging Group, University of Oulu, Oulu, Finland; 3 Turku School of Economics, University of Turku, Turku, Finland; Potsdam Institute for Climate Impact Research, GERMANY

## Abstract

What mechanism links climate change and social change? Palaeoanthropological analysis of human remains suggests that abrupt climate change is linked to societal restructuring, but it has been challenging to reliably identify the exact mechanisms underlying this relationship. Here we identify one potential mechanism that can link climate to behavior change, and underpins many of the reported findings on social restructuring. Specifically, we show that daily weather is linked to human *planning* behavior, and this effect is moderated by climate. Our results demonstrate that as weather gets colder, humans increase their planning in cold regions and decrease planning in warm regions. Since planning has previously been linked to group efficiency, cooperation, and societal organization, our work suggests *planning* is one mechanism that can link climate change to societal restructuring.

## Introduction

How does climate change shape human culture and society? Evidence suggests that the answer is related to human physiological and psychological change caused by the *situated weather*, which, in the long term, is believed to affect local human behavioral patterns to form culture-shaping habits or personality. In this study, we refer to climate as the long-term pattern of average weather conditions of a region, season as the annual changes, and weather as the daily changes observed in a region. Evidence has associated weather conditions, like temperature, sunshine, humidity and wind speed, to the changes of human pathology [1], mood and cognition status [2–4], as well as the tendency for crime [5,6] and suicides [7,8]. However, such research on daily weather changes does not address how humans’ situated response to weather evolves into a more persistent pattern of behavior and ultimately culture. For instance, a paradox is that if daily weather changes lead to a particular behavior (warm weather leads to violence) that in turn becomes a regional culture (people tend to be more violent), this behavior pattern will eventually perpetuate independently (e.g. these people become more violent than others independent of weather), thus conflicting with the original assumption. This limitation has partly led to a decline of environmental determinism in explaining climatic effect on human society since 1940s. However, even if scientists remain unclear on how climate change alters human society, social scientists are increasingly discovering new evidence linking weather and human behaviors, such as sunshine and rainfall to stock buying [9–11] and consumer decisions [12–14].

Interestingly, recent advances in palaeoanthropological analysis on human remains have offered new evidence to justify climatic influence on human society on a macro-level [15–18]. Based on the datasets collected from different periods of human history across different continents, previous research has found that abrupt climate change is linked to societal restructuring [15–18], and climatic change toward warmer temperature and extreme rainfall is associated with the events of human violence and collapse of civilizations [19]. However, despite the availability of this new and robust evidence, it remains unclear how macro-level climate affects individual behavioral to form culture on a micro level. The mechanism linking macro-level climatic influence to micro-level daily human activities remains unknown. Therefore, identifying a mechanism that links climate and weather to societal change is a research priority in science [19].

Unlike environmental determinism that regards human behavior as a passive consequence of the long-term effects of weather, we argue for another possibility: that humans can adapt their behavior to weather based on their knowledge of climate, and in doing so linking weather and climate to societal change.

To this end, we report a large-scale analysis to detect the possible moderating effects of a region’s climate on daily human *planning* behavior through the use of big data. Planning is linked to individual actions [20], group cooperation [21,22], and societal organization [23,24]. In regions with adverse climate, such as extreme heat or extreme cold, planning would have been a key survival skill for our early ancestors. Thus, understanding the relationship between climate and planning can provide important insights for interpreting the little understood link between increase to temperature, societal unrest, and violence. However, it is challenging to reliably study human planning in relation to climate, because this requires: i) a method to observe and measure planning with external validity, ii) a homogeneous population, and iii) climatic diversity.

Our study overcomes these three challenges by analysing the patterns of service coupons sale across China. We analysed data from Lashou, a Groupon-like website in China that sells coupons for local services such as restaurants, cafes, hairdressers, cinemas, hotels and KTVs (see S1 Text section 1). While typical retail websites sell items that are conveniently delivered to one’s doorstep, Lashou sells coupons that are valid during a specific period and for a specific local business. Therefore, a transaction on Lashou strongly indicates planning response: it reflects a person’s explicit commitment, vested through an up-front financial cost, to visit a particular local business within a specified timeframe in the near future. Furthermore, because the service is available to local Chinese businesses only, it is used by customers in Mainland China who form a genetically homogeneous population [25]. Finally, our data covers 28 cities across China, which exhibit major climatic differences due to their diverse geography.

## Data and Method

During a 16-month period we recorded 20.5 million transactions publicly announced on Lashou, grossing 1.2 billion CNY (145 million EUR, 201 million USD) across the 28 cities (see S1 Text sections 2 and 10). For each transaction we registered the date and time, price, city, and category of the business providing the service. We also recorded from Wunderground.com daily weather data for all 28 cities as provided by the international airports at each of those cities (see S1 Text section 3). Finally, we used official demographic data provided for each city by the central Chinese government (see S1 Text section 4).

We use Kawamura’s Discomfort Index (DIK) [26] to quantify the daily temperature and humidity in our sample, based on the formula:

$$\text{DIK}=\;0.99\;*\text{ T }+\;0.36\;*\;{\text{T}}_{\text{d}}+\;41.5$$

T is the mean air temperature (°C)

T_d_ is mean dew point temperature (°C)

Using DIK we classify each day of each city into one of three thermal comfort zones: cold (DIK< = 60), comfortable (60<DIK< = 75), and hot (DIK > 75). The set of dates in the cold zone are defined as the “winter” for each city. We also applied a log transformation to the total daily Lashou transaction value per city to achieve normal distribution. We proceed to analyse the direct effects of DIK on daily transactions, which we argue reflect the level of planning exhibited by residents of each city. The level of sunshine, measured by cloud cover, has been widely reported to alter human decision making and behaviour through altering human mood [10,11], and so we control for it.

### The effect of weather and socio-structural factors on planning behaviour

**Fig 1:**
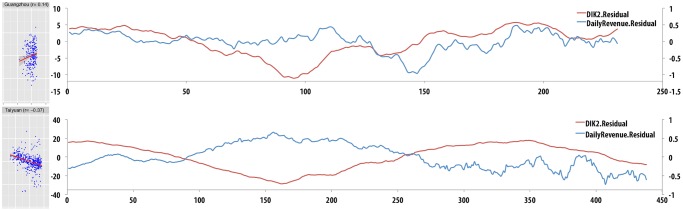
Correlation between temperature and planning activities. Scatterplots show the residuals for DIK (x-axis) versus revenue (y-axis) for each day in our dataset. Each scatterplot shows the data for a single city in our dataset, and reports the correlation coefficient. For each regression line we highlight the 95% CIs. The analysis controls for the effect of cloud cover for each data point. The line graphs show a detailed view of the weather and revenue data for the same 2 cities (Guangzhou and Taiyuan) over time.

We tested separately the effect of DIK on planning behaviour during comfortable-hot seasons (DIK > 60), and comfortable-cold seasons (DIK < = 75). A partial correlation analysis between daily DIK and transformed transaction values controlling for the effect of cloud-cover is performed for each city through the use of pcor.test function in R [27]. The analysis for comfortable-hot seasons did not provide significant results (see S1 Text section 5). The analysis for comfortable-cold seasons showed that 24 cities report significant negative effects (p < 0.05), 2 cities report significant positive effects (Fig 1), while 2 are not significant (see S1 Text section 6). The effects for 12 of these cities are highly significant (p < 0.001).

These significant results suggest that cities respond differently as weather transitions between cold and comfortable seasons: people plan more as the temperature drops in some cities, while in other cities people plan less. We proceed to investigate whether socio-structural or geographical factors can explain these opposite behaviours.

One possible explanation may relate to differences in cities’ societal structures, which lead to regional differences in planning response to cold weather. Therefore, we tested major societal structure variables, including the GDP, population, and GDP per capita (see S1 Text section 7). None of these variables significantly explain the tendency we identified, thus rejecting the socio-structural influence on people’s adaptation of planning response to cold weather.

### Explaining climatic influence on daily human behaviour

We next test whether these regional differences can be attributed to climatic or geographic conditions. We investigated three factors attributed to each city: the length of winter (in days), the coldest temperature recorded in our 16-month dataset, and latitude. These three are linked since Chinese cities at higher latitude tend to have colder and longer winters.

**Fig 2:**
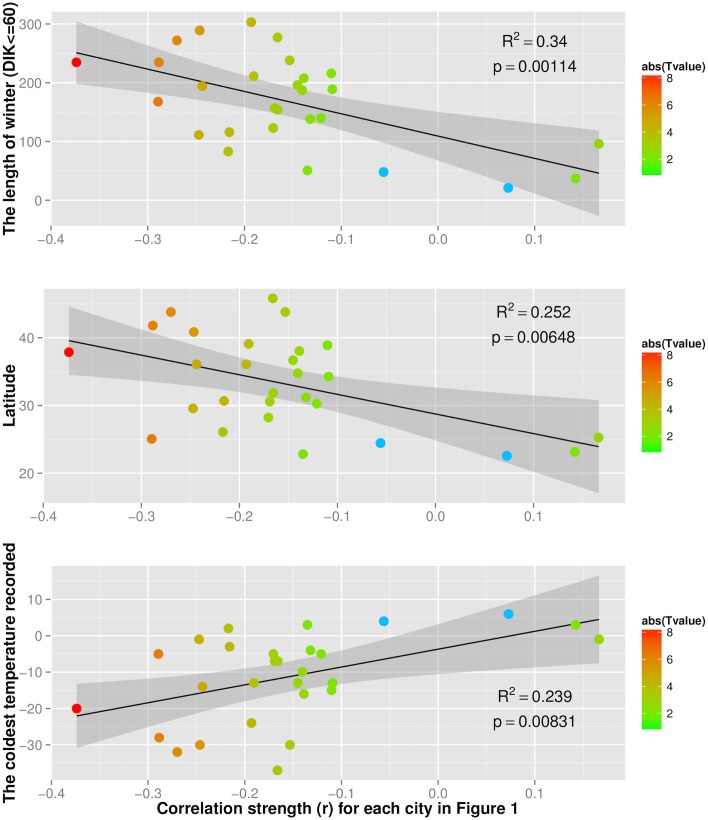
The effect of winter length (number of days) on planning activities across 28 cities. Each point represents a city in our dataset. For each city we indicate the length of winter defined as the number of days with DIK < = 60 (y-axis), the correlation strength as reported in S1 Text figure 1 (x-axis), and the absolute T-value of the correlation (color scale). Blue points represent the cities reporting no significant correlation. The grey area shows the 95% CIs.

**Fig 3:**
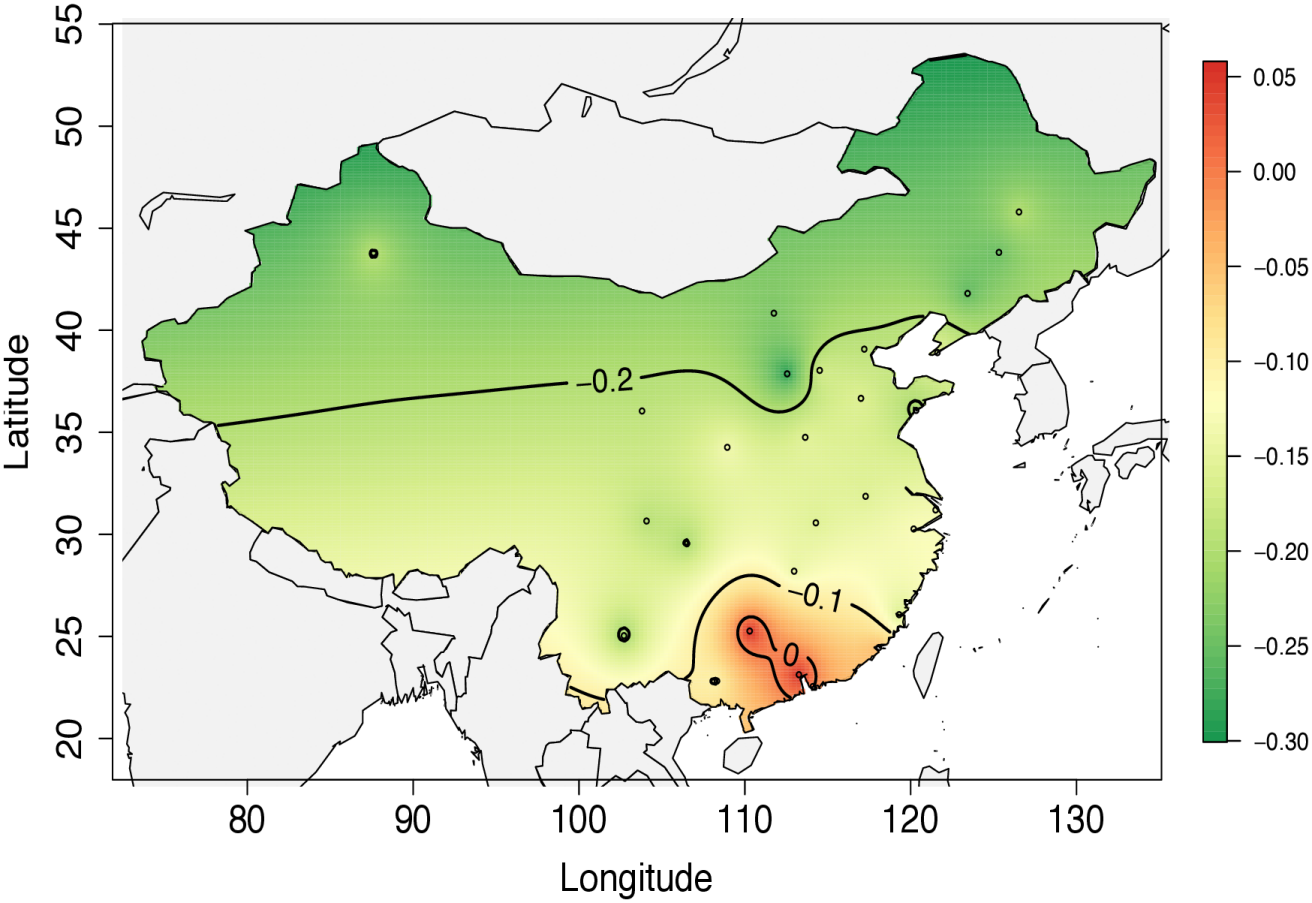
Geographic and climatic influence on human planning response. This map of Mainland China uses a Gaussian process regression (kriging) to visualize the geospatial distribution of the correlation values reported in S1 Text figure 1. We note that cities with a positive correlation are located near the south. The region in low latitude and close to the sea has a warmer climate than the region in high latitude and far from the sea. The black line with numeric value depicts the effect of temperature change to human planning activities.

Our results indicate that residents tend to plan more in cold weather when their city has longer winters (Pearson’s ρ = -0.583, p = 0.0011, 95% CI: -0.7851 to -0.2679) (Fig 2), colder climate (Pearson’s ρ = 0.489, p = 0.0083, 95% CI: 0.1415 to 0.7289), and higher latitude (Pearson’s ρ = -0.502, p = 0.0065, 95% CI: -0.7371 to -0.1588) (Fig 3). These results are robust: they hold even when we consider those days when DIK is within a common denominator band across all cities (47.95< = DIK< = 75) (see S1 Text section 8). Based on our results, if the long-term mean temperature (i.e. climate) of a city increases by 1 degree, its length of winter will decrease by 18.7 days and people’s planning tendency in cold weather will decrease by 1.6%.

The planning tendencies we have observed are consistent with the hypothesis that human planning behaviour has adapted to increase survival rate in cold climate. We found that as weather becomes colder, planning increases in cold regions while it decreases in hot regions. (No significant patterns were found for transitions to hot weather). It is unclear whether this propensity is genetically rooted in human survival during the ice age, or is culturally preserved. However, this finding provides a new way to explain previous research findings on weather’s influence on human behaviour.

### Validation of findings on a global scale

We subsequently tested our hypothesis on a global scale: using a second independent dataset that reflects planning response of individuals across 21 cities in 15 nations over a 4-year period. We expected that in cities with cold climate the planning behaviour of residents increases as the weather gets colder, while in cities with hot climate the planning behaviour of residents decreases as the weather gets colder.

To test our hypothesis we analysed data from Alexa.com on the number of online visitors between 2010 and 2013 to websites that provide regional public transport scheduling information (see S1 Text section 9). Our assumption is that a visit to such a website is a strong indicator of planning behaviour, and therefore we argue that as a population’s tendency to plan increases then we would observe increased visits to these transport scheduling websites. Climate data for each city was collected from Worldweatheronline.com. Because Alexa.com only provides the traffic data of top 100.000 independent websites in popularity, this limits our sample size to a selection of 21 cities.

The analysis of the data proceeded as follows. Comfortable months were defined as those months with average high temperature between 20 and 24°C. At most 4 comfortable months are included into the analysis with average high monthly temperature close to 24°C. The selection of 20–24°C was based on the characterization of the range 22–24°C as comfortable [28], and we slightly broaden this range to be 20–24 in order to enlarge the size of comfortable months in some cities and facilitate further analysis. We also consider cold months as those 3 months with the lowest average high temperature that is below 20 degrees. If two months have the same average high temperatures, the one with lower average low temperature is selected. December was excluded from analysis of traffic data in western countries to avoid the possible effect of Christmas. February is not included in the analysis for Asian countries for similar reasons.

Based on the comfortable and cold months identified, we calculated the mean value of their daily traffic and performed an ANOVA test to compare their mean values (see S1 Text section 9). We report the traffic difference divided by the average traffic of comfortable months. For cities with an insignificant difference we assign the value of 0 for the difference.

$$\text{Traffic difference }=\;\left({\text{Mean}}_{\text{comfortable}-\text{month traffic}}–{\text{ Mean}}_{\text{cold}-\text{month traffic}}\right)\;/{\text{ Mean}}_{\text{comfortable}-\text{month traffic}}$$

**Fig 4:**
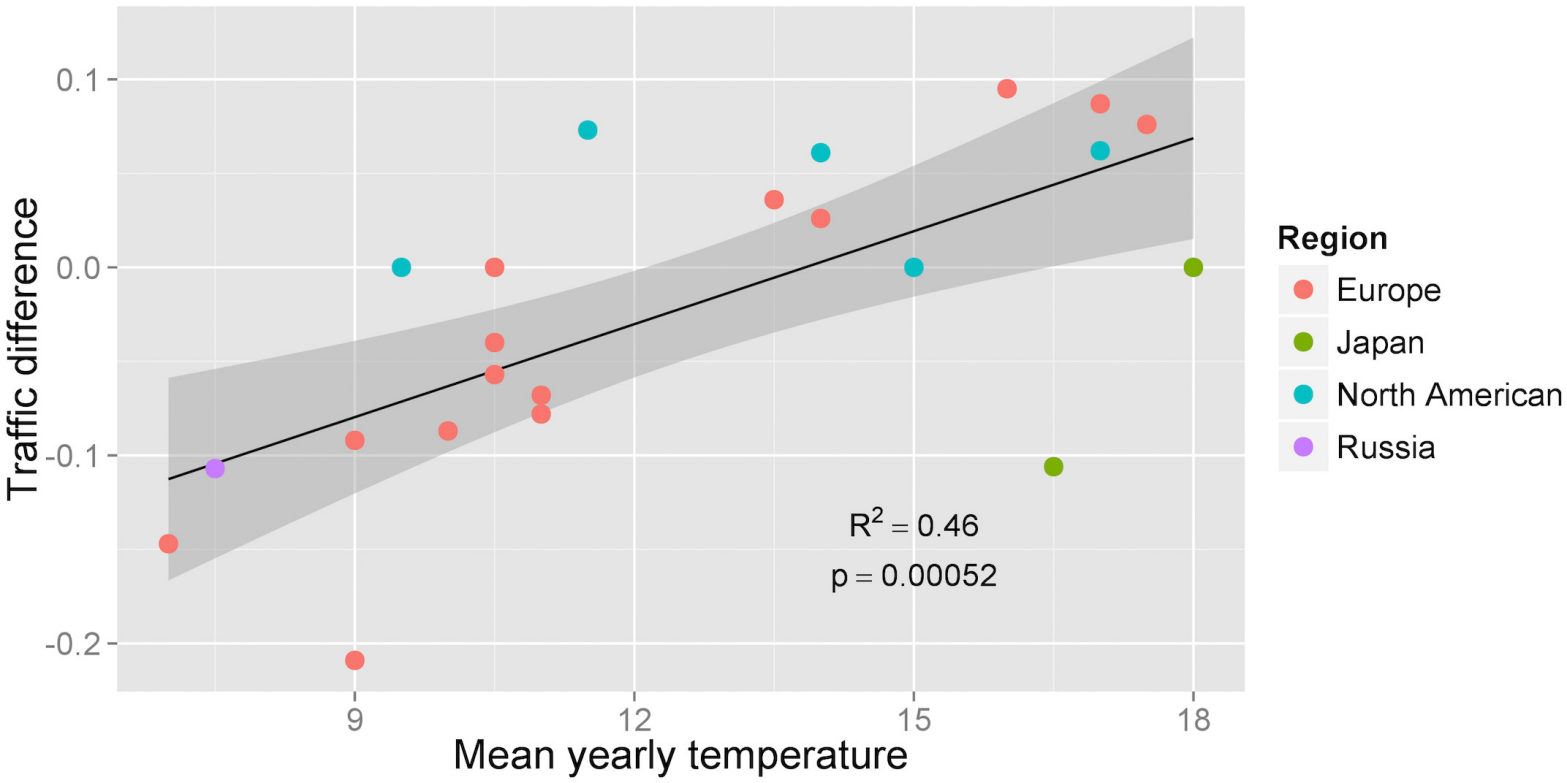
Correlation between average yearly temperature and traffic difference (between comfortable and cold months) for public transport websites. Each data point represents a city.

The results support our hypothesis: residents of cities with cold climate increase their visits to he public transport websites during cold months, and residents of cities with hot climate decrease their visits to the public transport websites during cold months (Pearson’s ρ = 0.703, p = 0.0004, 95% CI: 0.3907 to 0.8708) (Fig 4). To control our findings against other types of websites that do not reflect planning response, we performed the *same analysis* for the *same cities* but considering local government websites, which we expect do not relate to planning behaviour. Our analysis showed no significant effect of climate on the use of those websites (Pearson’s ρ = -0.024, p = 0.917, 95% CI: -0.4510 to 0.4118). These findings lend further support for our hypothesis on climatic effects on planning behaviour.

## Discussion

Social scientists are increasingly discovering new evidence linking weather and human behavior, such as sunshine and rainfall to stock buying [9–11] and consumer decisions [12–14]. Similarly, paleoanthropologists have shown that abrupt climate change is linked to societal restructuring [15–18], and climatic change toward warmer temperature and extreme rainfall is associated with the events of human violence and collapse of civilizations [19]. Our work suggests that humans’ *planning* behaviour may be the link that can reconcile these two sets of previous findings.

Our analysis of planning behaviour—as reflected in the sales of service coupons in China, or visits to public transportation websites across the world—shows that long-term climate primes our planning response to daily weather. We argue that these findings reconcile weather effects on behaviour and climatic effects on societal change. Planning has been linked to individual actions [20], group cooperation [21,22], and societal organization [23,24]. We expect that in regions with extreme cold, planning would have been a key survival skill for our early ancestors. Thus, our work in identifying the relationship between long-term climate, daily weather and planning is important helping us interpret why increase to temperature has led to societal unrest, and violence. A decrease in planning response can arguably result in lower productivity for individuals, weaker coordination for groups, and reduced social cohesion. We argue, therefore, that these in turn are likely to contribute to social unrest and restructuring linked to climate change [19].

It could be argued that our findings merely reflect browsing habits of modern society. For instance, is it the case that when the weather gets too cold people stay home and browse the web? Our findings suggest otherwise. Our analysis of the traffic data of municipal websites contradicts this assertion, since the analysis shows no link between regional climate and visits to such websites. Thus, it is not the case that our data merely reflects increased Internet usage during adverse weather.

Furthermore, it could be argued that our findings simply reflect consumer habits. For instance, is it the case that when the weather gets too cold in cold places, consumers possibly “take action”? This assertion is not supported by our data either, because we show that in cities with warm climate, when the weather gets cold they actually reduce their online purchases of coupons.

## Conclusion

Findings from a growing corpus of scientific evidence across multiple disciplines suggest that a change in the environment would exert considerable influence on human behaviour. Climate, as a dominating environmental factor, has been widely reported to be a factor of human genetic and societal evolution, and, as shown in our study, is still relevant today. Understanding the mechanism linking long-term climate to human behavioural change is vital, and we show that planning response is key to linking these two.

Further, our results provide a new alternative to explain the mechanism underlying climatic effects on human society. Rather than regarding human society as a passive outcome of long-term effect of climate, our results suggest another possibility: people proactively adapt their behaviour to situated weather based on their knowledge of the long-term characteristics of the climate. In other words, people respond to current weather based on their expectation on the future conditions of weather and this process is proactive.

## Ethics statement

The collection and analysis of data from Lashou.com, Alexa.com, Wunderground.com, and Worldweatheronline.com adheres to Terms of Use for these services. The demographic data was made publicly available by the Chinese government. No personally identifiable data was collected or analysed in this study. No ethical approval was necessary for this study because it is based on public documents, registries and archived data.

## Supporting Information

S1 Text Supplementary information on data collection and analysis. (PDF)

## References

[1] Fleming JR (2006) The pathological history of weather and climate modification: Three cycles of promise and hype. The pathological history of weather and climate modification: Three cycles of promise and hype.

[2] Keller MC, Fredrickson BL, Ybarra O, Côté S, Johnson K, Mikels J, et al (2005) A Warm Heart and a Clear Head: The Contingent Effects of Weather on Mood and Cognition. Psychological Science 16: 724–731. 16137259 10.1111/j.1467-9280.2005.01602.x

[3] Howarth E, Hoffman MS (1984) A multidimensional approach to the relationship between mood and weather. British Journal of Psychology 75: 15–23. 6704634 10.1111/j.2044-8295.1984.tb02785.x

[4] Nastos PT, Paliatsos AG, Tritakis VP, Bergiannaki A (2006) Environmental discomfort and geomagnetic field influence on psychological mood in Athens, Greece. Indoor and Built Environment 15: 365–372.

[5] Cohn EG (1990) Weather and Violent Crime: A Reply to Perry and Simpson, 1987. Environment and Behavior 22: 280–294.

[6] Cohn EG (1990) Weather and crime. British journal of criminology 30: 51–64.

[7] Stoupel E, Abramson E, Sulkes J (1999) The effect of environmental physical influences on suicide: How long is the delay? Archives of Suicide Research 5: 241–244.

[8] Barker A, Hawton K, Fagg J, Jennison C (1994) Seasonal and weather factors in parasuicide. The British Journal of Psychiatry 165: 375–380. 7994509 10.1192/bjp.165.3.375

[9] Kamstra MJ, Kramer LA, Levi MD (2003) Winter blues: A SAD stock market cycle. American Economic Review: 324–343.

[10] Saunders EM Jr (1993) Stock prices and Wall Street weather. American Economic Review 83: 1337–1345.

[11] Hirshleifer D, Shumway T (2003) Good day sunshine: Stock returns and the weather. The Journal of Finance 58: 1009–1032.

[12] Parsons AG (2001) The association between daily weather and daily shopping patterns. Australasian Marketing Journal (AMJ) 9: 78–84.

[13] Gardner MP (1985) Mood states and consumer behavior: a critical review. Journal of Consumer Research: 281–300.

[14] Murray KB, Di Muro F, Finn A, Popkowski Leszczyc P (2010) The effect of weather on consumer spending. Journal of Retailing and Consumer Services 17: 512–520.

[15] Kennett DJ, Breitenbach SF, Aquino VV, Asmerom Y, Awe J, Baldini JAU, et al (2012) Development and disintegration of Maya political systems in response to climate change. Science 338: 788–791. doi: 10.1126/science.1226299 23139330 10.1126/science.1226299

[16] Núñez L, Grosjean M, Cartajena I (2002) Human occupations and climate change in the Puna de Atacama, Chile. Science 298: 821–824. 12399589 10.1126/science.1076449

[17] Polyak VJ, Asmerom Y (2001) Late Holocene climate and cultural changes in the southwestern United States. Science 294: 148–151. 11588259 10.1126/science.1062771

[18] DeMenocal PB (2001) Cultural responses to climate change during the late Holocene. Science (New York, NY) 292: 667–673. 11303088 10.1126/science.1059827

[19] Hsiang SM, Burke M, Miguel E (2013) Quantifying the influence of climate on human conflict. Science 341: 1235367 doi: 10.1126/science.1235367 24031020 10.1126/science.1235367

[20] Lynch JG Jr, Netemeyer RG, Spiller SA, Zammit A (2010) A generalizable scale of propensity to plan: the long and the short of planning for time and for money. Journal of Consumer Research 37: 108–128.

[21] Whitney JC, Smith RA (1983) Effects of group cohesiveness on attitude polarization and the acquisition of knowledge in a strategic planning context. Journal of Marketing Research: 167–176.

[22] Kartez JD (1991) Planning for cooperation in environmental dilemmas. Journal of Planning Literature 5: 226–237.

[23] Lindsay WM, Rue LW (1980) Impact of the organization environment on the long-range planning process: A contingency view. Academy of Management Journal 23: 385–404.

[24] Nkomo SM (1987) Human resource planning and organization performance: An exploratory analysis. Strategic Management Journal 8: 387–392.

[25] Oota H, Kitano T, Jin F, Yuasa I, Wang L, Ueda S, et al (2002) Extreme mtDNA homogeneity in continental Asian populations. American journal of physical anthropology 118: 146–153. 12012367 10.1002/ajpa.10056

[26] Kawamura T (1965) Distribution of discomfort index in Japan in summer season. J Meteorol Res 17: 460–466.

[27] Kim S (2012) ppcor: Partial and Semi-partial (Part) correlation. The Comprehensive R Archive Network website. URL http://CRAN.R-project.org/package=ppcor. Accessed: 0–9.

[28] Pfafferoot JU, Herkel S, Kalz D, Zeuschner A (2007) Comparison of low-energy office buildings in summer using different thermal comfort criteria. Energy and Buildings 39: 750–757.

