# Correction: Climatic Effects on Planning Behavior

**DOI:** 10.1371/journal.pone.0131954

**Published:** 2015-06-25

**Authors:** Yong Liu, Vassilis Kostakos, Hongxiu Li

There are errors in Figures 2 and 3 of S1 Text. Please view the correct S1 Text below.

## Supporting Information

S1 Text Supplementary information on data collection and analysis. (DOCX)
