# Supplementary material for: Climatic Effects on Planning Behavior
Source: PLoS One. 2015 May 19;10(5):e0126205. doi: 10.1371/journal.pone.0126205 (PMC4437775; doi:10.1371/journal.pone.0126205)
Supplement: S1 Text — (PDF) [file pone.0126205.s001.pdf]

Supplementary Information

Table of Contents

Section 1: A description of Lashou.com 2

Section 2: Data Collection from Lashou.com 2

Section 3: Weather data 3

Section 4: City Demographic data 3

Section 5: The effect of DIK in comfortable-hot weather conditions 4

Section 6: The effect of DIK in comfortable-cold weather conditions 5

Section 7: The possible moderating effects of major societal structure variables on daily weather's influence on planning activities 6

Section 8: The effect of DIK within a common denominator band across all cities6

Section 9: Climatic effects on the popularity of public transportation sites and government sites 8

Section 10: Server failure, outliers and lost data 12

Section 1: A description of Lashou.com

Similar to Groupon in the US, Lashou Group Inc. (Lashou) is an online group-buying site that offers products and services at significantly reduced prices on the condition that a minimum number of buyers would make the purchase. Founded in 2009, Lashou offers discounted offerings primarily in the categories of food and entertainment, health and beauty, and travel and hospitality in over 400 Chinese cities. Typically, a consumer needs to purchase a coupon on the website and show/use the coupon at the local business provider within a predefined period of time. In 2013, the site declared that it has over 3 million registered customers with 30 million average monthly visits[1]. In addition to its online sale platform, the company also distributes its product information through its API to various coupon-searching engines.

Section 2: Data Collection from Lashou.com

We ran a script to automatically collect API data given by Lashou.com and save it to our database between 2012-07-14 and 2013-11-14. The script is run automatically during midnight (starting at 23:30) every day. The API data includes product id, product title, url, start time, planned end time, original price, (current) price, category, city, image and the number of sold coupons. Because the amount of sold coupons is updated in real-time, we can infer the daily number of coupons sold by subtracting the number for date N-1 from date N.

In this manner we collected over 27 million database rows of transactions for 565011 items sold at 521 different Chinese cities or towns. Three categories of products are excluded from analysis, including

- lucky draw (price = 0, N = 274);
- products sold across all cities (N = 47397);
- products sold in multiple cities, such as chain hotels (189915 items from actually 9368 products).

For those sold in multiple cities, it is impossible to calculate their sale in each specific city, and we therefore excluded these products from our city-level analysis. We did retain a single record for such items when analysing the overall transaction volume for the site. As a result, transaction details of 327425 products are retained for conducting city-level analysis.

The data analysis further focused on 28 Mainland Chinese cities due to the restrictions of weather data collection, as specified next in Section 3.

[1] http://b2b.toocle.com/detail--6146414.html

Section 3: Weather data

We collected weather data from the weather website (wunderground.com), which obtains weather data from the weather stations of International airports in China. Through the website we collect a number of weather attributes: air temperature, humidity, dew point temperatures, wind speed, visibility, precipitation and cloud cover.

Precise weather information (e.g. cloud-cover, humidity and temperature) can only be collected from 29 major Mainland Chinese cities that have International airports. The capital of China, Beijing, is further removed from analysis due to its instability in resident population during winter. Specifically, Beijing has the biggest amount of university students and city immigrants in China. About 37.4% of Beijing's population is city immigrants (those living in Beijing for over 6 months) in 2012[2], without counting millions of those living in Beijing less than 6 months. During the month of spring festival, 9 million left Beijing for family reunions in 2013[3].

Of the 28 cities we retain for further analysis, we note that for 9 cities the weather stations did not report precipitation data. Dew point temperature is a product of both air temperature and humidity (1). Cloud-cover (out of 8) means the fraction of cloud cover in a given day: 0 means sky completely clear while 8 means sky completely cloudy. The less cloud cover, the more sunshine the day will have.

Section 4: City Demographic data

City demographic information is collected from official China Statistical Yearbook 2013. From the book, we obtain over 100 different attributes for each city. The major attributes that we have included in the analysis are:

- GDP of city
- Population of city
- GDP per capita of city
- Population density of city
- Percentage of primary industry in GDP of city
- Number of theatres in city
- Amount of mobile phone users in city
- Amount of Internet users in city
- Industrial wastewater emissions
- Number of hospitals in city

[2] http://politics.gmw.cn/2013-05/29/content_7783839.htm
[3] http://usa.chinadaily.com.cn/china/2013-02/10/content_16217854.htm

Section 5: The effect of DIK in comfortable-hot weather conditions

Table 1 shows the results of partial Pearson's correlations between DIK and planning activities across 28 cities during comfortable-hot weather conditions (DIK>60), after controlling the effect of cloud-cover.

City Name	ρ	p-value	t-value	Winter days	Latitude	Longitude	Minimal Temperature
Guilin	**0,234**	**7,01E-06**	**4,493**	96	25.27	110.29	-1
Guangzhou	0,048	0,33376783	0,967	37	23.13	113.26	3
Shenzhen	**0,100**	**0,03729499**	**2,083**	21	22.55	114.1	6
Xiamen	0,003	0,95981723	0,050	48	24.46	118.08	4
Xi'an	-0,064	0,30134081	-1,034	189	34.27	108.94	-13
Dalian	0,119	0,06961282	1,814	216	38.91	121.61	-15
Hangzhou	0,016	0,77649939	0,284	140	30.27	120.18	-5
Shanghai	-0,067	0,2414219	-1,171	138	31.2	121.5	-4
Nanning	**0,133**	**0,00735931**	**2,680**	51	22.82	108.32	3
Shijiazhuang	-0,045	0,48771165	-0,694	208	38.04	114.51	-16
Zhengzhou	-0,039	0,53214414	-0,625	187	34.76	113.64	-10
Jinan	-0,015	0,80681708	-0,245	196	36.67	117	-13
Urumqi	-0,048	0,49132447	-0,688	238	43.8	87.61	-30
Hefei	-0,010	0,86336626	-0,172	154	31.87	117.28	-7
Harbin	-0,076	0,31999808	-0,994	277	45.8	126.53	-37
Wuhan	**0,213**	**0,00019105**	**3,731**	157	30.57	114.28	-7
Changsha	-0,014	0,80779856	-0,243	123	28.2	112.97	-5
Tianjin	0,043	0,50720703	0,663	211	39.08	117.2	-13
Lanzhou	-0,113	0,17224579	-1,365	303	36.05	103.8	-24
Chengdu	0,008	0,88449737	0,145	116	30.66	104.07	-3
Fuzhou	**-0,130**	**0,01225907**	**-2,505**	83	26.07	119.3	2
Qingdao	**0,180**	**0,00367292**	**2,905**	194	36.08	120.33	-14
Hohhot	**0,162**	**0,04011507**	**2,053**	289	40.84	111.75	-30
Chongqing	-0,018	0,73856351	-0,334	111	29.55	106.51	-1
Changchun	0,095	0,20484884	1,268	272	43.82	125.32	-32
Shenyang	**0,149**	**0,02831218**	**2,193**	235	41.81	123.43	-28
Kunming	-0,110	0,06723192	-1,830	168	25.04	102.71	-5
Taiyuan	-0,026	0,70288704	-0,381	235	37.87	112.55	-20

Table 1. The strength of comfortable-hot weather's influence on planning activities (significant correlations are annotated in bold). For each city we show: Pearson's partial correlation between daily revenue and DIK (ρ), p-value and t-value of the test, the length of winter defined as the number of cold days (DIK <=60), the latitude and longitude, and the minimal temperature (Celsius) recorded in our data. The Benjamini & Hochberg (3) procedure was followed to control the false discovery rate set at Q=0.20.

Section 6: The effect of DIK in comfortable-cold weather conditions

Table 2 shows the results of partial Pearson's correlations between DIK and planning activities across 28 cities during comfortable-cold weather conditions (DIK<=75), after controlling the effect of cloud-cover.

City Name	ρ	p-value	t-value	Winter days	Latitude	Longitude	Minimal Temperature
Guilin	**0,166**	**0.004182**	**2.864**	96	25.27	110.29	-1
Guangzhou	**0.142**	**0.028417**	**2.191**	37	23.13	113.26	3
Shenzhen	0.072	0.282655	1.074	21	22.55	114.1	6
Xiamen	-0.056	0.369161	-0.898	48	24.46	118.08	4
Xi'an	**-0.109**	**0.035839**	**-2.099**	189	34.27	108.94	-13
Dalian	**-0.110**	**0.02528**	**-2.237**	216	38.91	121.61	-15
Hangzhou	**-0.121**	**0.032627**	**-2.137**	140	30.27	120.18	-5
Shanghai	**-0.132**	**0.018984**	**-2.346**	138	31.2	121.5	-4
Nanning	**-0.135**	**0.035064**	**-2.108**	51	22.82	108.32	3
Shijiazhuang	**-0.139**	**0.00664**	**-2.714**	208	38.04	114.51	-16
Zhengzhou	**-0.140**	**0.0073**	**-2.683**	187	34.76	113.64	-10
Jinan	**-0.145**	**0.005394**	**-2.782**	196	36.67	117	-13
Urumqi	**-0.153**	**0.001161**	**-3.248**	238	43.8	87.61	-30
Hefei	**-0.166**	**0.002637**	**-3.007**	154	31.87	117.28	-7
Harbin	**-0.166**	**0.000407**	**-3.535**	277	45.8	126.53	-37
Wuhan	**-0.169**	**0.00188**	**-3.109**	157	30.57	114.28	-7
Changsha	**-0.170**	**0.002567**	**-3.015**	123	28.2	112.97	-5
Tianjin	**-0.191**	**0.000193**	**-3.728**	211	39.08	117.2	-13
Lanzhou	**-0.193**	**3.18E-05**	**-4.160**	303	36.05	103.8	-24
Chengdu	**-0.216**	**4.43E-05**	**-4.084**	116	30.66	104.07	-3
Fuzhou	**-0.217**	**0.000236**	**-3.677**	83	26.07	119.3	2
Qingdao	**-0.244**	**2.12E-06**	**-4.742**	194	36.08	120.33	-14
Hohhot	**-0.246**	**8.11E-08**	**-5.365**	289	40.84	111.75	-30
Chongqing	**-0.247**	**6.80E-06**	**-4.500**	111	29.55	106.51	-1
Changchun	**-0.270**	**3.91E-09**	**-5.888**	272	43.82	125.32	-32
Shenyang	**-0.288**	**1.12E-09**	**-6.092**	235	41.81	123.43	-28
Kunming	**-0.289**	**2.15E-10**	**-6.350**	168	25.04	102.71	-5
Taiyuan	**-0.374**	**4.21E-17**	**-8.407**	235	37.87	112.55	-20

Table 2. The strength of cold weather's influence on planning activities (significant correlations are annotated in bold). For each city we show: Pearson's partial correlation between daily revenue and DIK (ρ), p-value and t-value of the test, the length of winter defined as the number of cold days (DIK <=60), the latitude and longitude, and the minimal temperature (Celsius) recorded in our data. The Benjamini & Hochberg (3) procedure was followed to control the false discovery rate set at Q=0.20.

Table 3 shows the relationship between the ρ values reported in Table 3 and long-term climate-related variables based on a Pearson's correlation test.

Regional climatic variable	ρ	t-value	p-value	95% CI
The number of cold days	-0.583	-3.656	0.00114	-0.7851, -0.2679
Latitude	-0.502	-2.961	0.00648	-0.7371, -0.1588
Minimal temperature in our dataset	0.489	2.857	0.00831	0.1415, 0.7289

Table 3. For all 28 cities we correlate certain regional climatic variables independently against the correlation values reported in Table 3.

Section 7: The possible moderating effects of major societal structure variables on daily weather's influence on planning activities

Table 4 summarizes the results of testing the possible moderating effects of society-structure variables (from Section 4). For all 28 cities we use Pearson's correlation to test each city attribute independently against the ρ values reported in Table 3.

City attribute	ρ	p-value
Population of whole city	-0.1620	0.4101
GDP of whole city	0.2327	0.2333
GDP per capita of whole city	0.3245	0.0919
Population density of whole city	0.2663	0.1707
Percentage of primary industry in GDP of whole city	0.1402	0.4766
Number of Theaters of whole city	-0.0842	0.6699
Amount of mobile phone users of whole city	0.3091	0.1095
Amount of Internet users of whole city	0.0095	0.9616
Industrial wastewater emissions	0.1236	0.5306
Amount of hospitals of whole city	-0.2481	0.2029

Table 4. Predicting the strength of cold weather's influence on planning activities through societal variables. Each city attribute is correlated with the ρ values reported in Table 3, and here we show the outcome of this test.

Section 8: The effect of DIK within a common denominator band across all cities

Table 5 shows of partial Pearson's correlation between DIK and planning activities across 28 cities for a specific subset of days when DIK is within a common band ($47.95 <= DIK <= 75$) that is common to all cities, after controlling the effect of cloud-cover. The low end of this common denominator band is 47.95, decided by the city (Shenzhen) that has the highest minimal DIK value across all cities.

As shown in Table 5, both positive and negative correlations between DIK and planning activities have been found while the regression line remains significant (Pearson's ρ = -0.497, p = 0.007, 95% CI: -0.7342 to -0.1526) (Figure 1.). The regression line remains significant both when we set the correlation of the cities with insignificant results to be 0 (Pearson's ρ = -0.48, p = 0.0096, 95% CI: -0.7239 to -0.1309) or remove them (Pearson's ρ = -0.622, p = 0.023, 95% CI: -0.8737 to -0.1087).

City Name	ρ	p-value	t-value	Winter days	Latitude	Longitude
Guangzhou	**0,140718**	**0,030396**	**2,164888**	36	23,13	113,26
Guilin	**0,139054**	**0,019658**	**2,332804**	84	25,27	110,29
Dalian	**0,136114**	**0,025039**	**2,240799**	76	38,91	121,61
Shenyang	**0,129291**	**0,043392**	**2,019915**	66	41,81	123,43
Shenzhen	0,074083	0,274926	1,091789	20	22,55	114,1
Lanzhou	0,068144	0,266186	1,111889	121	36,05	103,8
Hohhot	0,061934	0,325553	0,98311	95	40,84	111,75
Changchun	0,04456	0,4763	0,712267	86	43,82	125,32
Qingdao	0,02001	0,758493	0,30746	74	36,08	120,33
Jinan	0,00711	0,911387	0,111289	82	36,67	117
Shijiazhuang	-0,00459	0,942408	-0,07224	81	38,04	114,51
Harbin	-0,02958	0,642514	-0,46419	82	45,8	126,53
Xi'an	-0,03517	0,562369	-0,57933	94	34,27	108,94
Xiamen	-0,04485	0,474307	-0,71549	47	24,46	118,08
Zhengzhou	-0,04599	0,458704	-0,74098	88	34,76	113,64
Urumqi	-0,05151	0,366955	-0,90219	105	43,8	87,61
Taiyuan	-0,05519	0,34326	-0,94774	94	37,87	112,55
Tianjin	-0,08343	0,190926	-1,30784	86	39,08	117,2
Shanghai	-0,10737	0,076512	-1,77129	97	31,2	121,5
Hefei	**-0,12642**	**0,038025**	**-2,07458**	99	31,87	117,28
Nanning	**-0,13624**	**0,034255**	**-2,11706**	49	22,82	108,32
Wuhan	**-0,14982**	**0,012289**	**-2,50373**	101	30,57	114,28
Hangzhou	**-0,15777**	**0,008909**	**-2,61555**	101	30,27	120,18
Changsha	**-0,16781**	**0,004464**	**-2,84337**	98	28,2	112,97
Chengdu	**-0,19207**	**0,000385**	**-3,54985**	103	30,66	104,07
Fuzhou	**-0,19745**	**0,000875**	**-3,32792**	82	26,07	119,3
Chongqing	**-0,22847**	**4,28E-05**	**-4,09182**	104	29,55	106,51
Kunming	**-0,23461**	**4,96E-07**	**-5,02792**	161	25,04	102,71

Table 5. The strength of cold weather's influence on planning activities of the same DIK interval (47.95<=DIK<=75) (significant correlations are annotated in bold). The Benjamini & Hochberg (3) procedure was followed to control the false discovery rate set at Q=0.20.

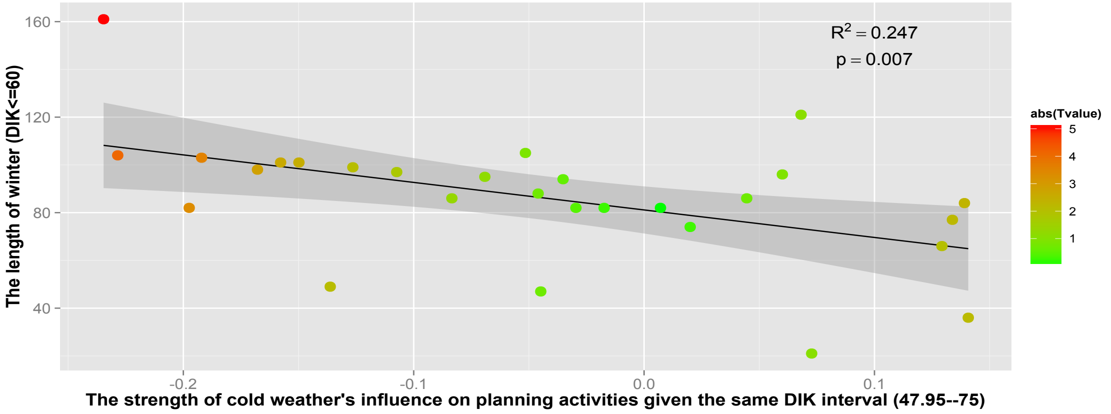

Figure 1. Visualisation of the data from Table 6. The x-axis denotes ρ, the y-axis denotes length of winter, and the color indicates t-value (absolute).

Section 9: Climatic effects on the popularity of public transportation sites and government sites

We seek to test our hypothesis regarding climatic effects on planning behaviour on a global scale. To do this we identify a number of cities internationally and collect data for their public transport websites and government websites. For each city we collected website traffic data from Alexa.com and weather data from worldweatheronline.com. We try to include as many cities as possible into the data analysis, but three conditions need to be met for a city to be included into the analysis:

1. Both the transport and government websites should be popular enough to make it to the top 100,000 sites of the world as reported by Alexa.com. In practice, this means that only major cities can be included in our analysis.
2. Both websites need to be independent and belong to different domains. In certain cities a single domain hosts both transport and government pages, and these cannot be included because Alexa does not provide the traffic data for sub-sites.
3. The city should have both comfortable and cold months. Otherwise, the city cannot be included into analysis.

The analysis of the data proceeded as follows. Comfortable months were defined as those months with average high temperature between 20 and 24°C. At most, 4 comfortable months are included into analysis with average high monthly temperature close to 24 °C. The selection of 20-24°C was based on the characterization of the range 22-24°C as comfortable (4). We slightly broaden this range to be 20-24 in order to enlarge the size of comfortable months in some cities and facilitate further analysis. We also consider cold months as those 3 months with the lowest average high temperature that is below 20 degree. If two months have the same average high temperatures, the one with lower average low temperature is selected. December was excluded from analysis of traffic data in western countries to avoid the possible effect of Christmas. February is not included in the analysis for Asian countries for similar reasons.

Traffic data was collected from Alexa, which provides data on the “Reach Per Million” of each website. This provides a rough percentage of the global population of Internet users that visits each website each day. We excluded the days that reported no traffic (“Reach Per Million” is null or 0). Outlier dates are also removed. This are defined as having traffic that is 3 standard deviations above or below the mean for a particular website, and should be less than 0.2% of rows of data statistically.

We further excluded data from particular cities. Oslo’s route planner website started to become popular since the middle of 2012. Therefore, we analyzed the traffic of this website since 2013-01-01. The Oslo government site starts to become popular at the end of 2012. Therefore we only include the year 2013 for its analysis. The traffic of Warsaw city government site in Alexa starts from 2011-08-01. Therefore we only include two years of data (2012-2013). The traffic of Hamburg public transportation and city government sites show a substantial drop at the beginning of 2013. Therefore, we only include 3 years of data (2010-2012) for analysis. The Madrid government website was not popular in 2010, therefore the analysis includes the data that starts from 2011-01-01.

Based on the comfortable and cold months identified, we calculated the mean value of their daily traffic and performed an ANOVA test to compare their mean values. We used the coding 0 for cold months and 1 for comfortable months, and tested whether the mean traffic differs significantly between these groups. We report the traffic difference divided by the average traffic of comfortable months. For cities with an insignificant difference we assign the value of 0.

Figure 2 visualises the relationship between the mean yearly temperature for each city (x-axis) and the traffic difference between comfortable and cold months for the public transport websites (Pearson's ρ = 0.678, p = 0.0005, 95% CI: 0.3594 to 0.8552). This data is shown in Table 6. Figure 3 shows the same analysis for government websites (Pearson's ρ = -0.029, p = 0.895, 95% CI: -0.4458 to 0.3967), which is shown in Table 7.

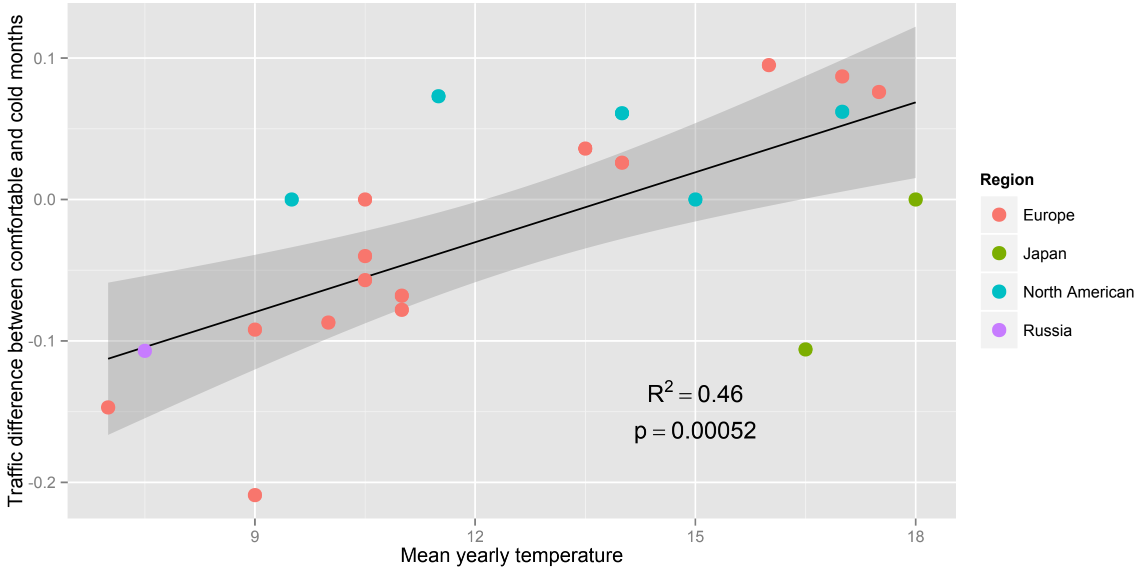

Figure 2. Correlation between mean yearly temperature and traffic difference for public transport websites. Each data point represents a city.

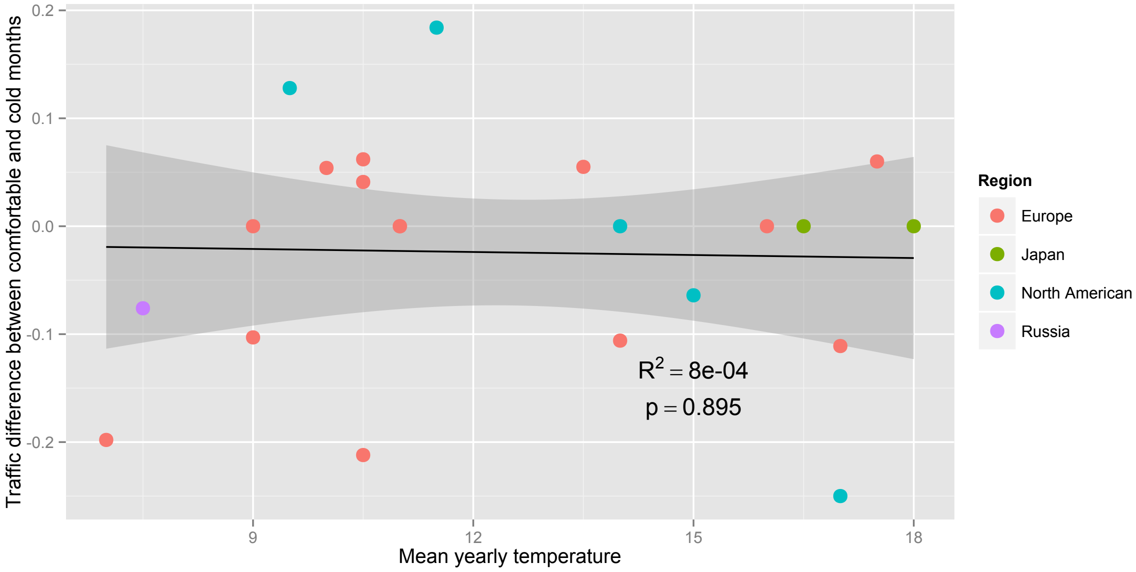

Figure 3. Correlation between mean yearly temperature and traffic difference for government websites. Each data point represents a city.

ID	City	Country	Website	Period of traffic data	Min average temp.	Cold months (<20°C)	Max average temp.	Comfortable months (20-24°C)	Mean of min & max average temp.	Traffic for comfortable months	Traffic for the cold months	Difference in traffic	p-value
1	Helsinki	Finland	hsl.fi	2010-01-01 (4 years)	-6	1;2;3	20	7	7.0	40.831	46.828	-0,147	0,0000040765
2	Moscow	Russia	engl.mosmetro.ru	2010-01-01 (4 years)	-9	1;2;11	24	6;7;8	7.5	24.443	27.066	-0,107	0,0001353848
3	Oslo	Norway	ruter.no	2013-01-01 (1 year)	-5	1;2;11	23	6;7;8	9.0	38.538	46.591	-0,209	0,0000313560
4	Stockholm	Sweden	sl.se	2010-01-01 (4 years)	-5	1;2;3	23	6;7;8	9.0	114.803	125.365	-0,092	0,0000074524
5	Toronto	Canada	ttc.ca	2010-01-01 (4 years)	-6	1;2;3	25	6;9	9.5	29.559	29.019	0,000	Insignificant
6	Munich	German	mvv-muenchen.de	2010-01-01 (4 years)	-4	1;2;11	24	6;7;8	10.0	118.421	128.694	-0,087	0,0000901303
7	Warsaw	Poland	ztm.waw.pl	2010-01-01 (4 years)	-4	1;2;11	25	5;6;8	10.5	69.283	72.039	-0,040	0,0083763080
8	Berlin	German	bvg.de	2010-01-01 (4 years)	-2	1;2;11	23	6;7;8	10.5	238.198	251.868	-0,057	0,0029677620
9	Copenhagen	Denmark	rejseplanen.dk	2010-01-01 (4 years)	-1	1;2;3	22	7;8	10.5	62.530	63.719	0,000	Insignificant
10	Hamburg	German	hvv.de	2010-01-01 2012-12-31	-1	1;2;3	23	6;7;8	11.0	122.968	131.289	-0,068	0,0000000047
11	Dublin	Ireland	dublinbus.ie	2010-01-01 (4 years)	2	1;2;10	20	7	11.0	22.548	24.302	-0,078	0,0130904400
12	Chicago	USA	transitchicago.com	2010-01-01 (4 years)	-6	1;2;3	29	5	11.5	24.895	23.067	0,073	0,0130140200
13	Paris	France	transilien.com	2010-01-01 (4 years)	2	1;2;11	25	5;6;9	13.5	89.075	85.833	0,036	0,0318861100
14	New York	USA	mta.info	2010-01-01 (4 years)	-2	1;2;3	30	5;10	14.0	254.367	238.964	0,061	0,0004937409
15	London	UK	tfl.gov.uk	2010-01-01 (4 years)	5	1;2;3	23	6;7;8;9	14.0	543.002	528.740	0,026	0,0398735000
16	Washington	USA	wmata.com	2010-01-01 (4 years)	-2	1;2;3	32	4;10	15.0	63.21311	64.36932	0,000	Insignificant
17	Istanbul	Turkey	iett.gov.tr	2010-01-01 (4 years)	3	1;2;3	29	5;10	16.0	31.661	28.645	0,095	0,0012287880
18	Tokyo	Japan	tokyometro.jp	2010-01-01 (4 years)	2	1;3	31	5;10	16.5	72.214	79.855	-0,106	0,0117180900
19	Los Angeles	USA	metro.net	2010-01-01 (4 years)	9	2;3	25	6;7;10;11	17.0	47.156	44.232	0,062	0,0001439345
20	Barcelona	Spain	tmb.cat	2010-01-01 (4 years)	5	1;2;3	29	5;10	17.0	30.4257	27.781	0,087	0,000259336
21	Madrid	Spain	metromadrid.es	2010-01-01 (4 years)	2	1;2;11	33	5;10	17.5	30.980	28.629	0,076	0,0010478220
22	Osaka	Japan	kotsu.city.osaka.lg.jp	2010-01-01 (4 years)	3	1;3	33	4;10	18.0	46.402	45.864	0,000	Insignificant

Table 6. A comparison of the traffic on public transport sites between comfortable and cold months. The p-value is obtained using an ANOVA test. The Benjamini & Hochberg (3) procedure was followed to control the false discovery rate set at Q=0.20.

ID	City	Country	Website	Period of traffic data	Min average temp.	Cold months (<20°C)	Max average temp.	Comfortable months (20-24°C)	Mean of min & max average temp.	Traffic for comfortable months	Traffic for the cold months	Difference in traffic	p-value
1	Helsinki	Finland	hel.fi	2010-01-01 (4 years)	-6	1;2;3	20	7	7.0	44.081	52.794	-0.198	0.0000000178
2	Moscow	Russia	mos.ru	2010-01-01 (4 years)	-9	1;2;11	24	6;7;8	7.5	97.774	105.231	-0.076	0.0213259800
3	Oslo	Norway	oslo.kommune.no	2013-01-01 (1 year)	-5	1;2;11	23	6;7;8	9.0	20.012	20.167	0	insignificant
4	Stockholm	Sweden	international.stockholm.se	2010-01-01 (4 years)	-5	1;2;3	23	6;7;8	9.0	55.707	61.424	-0.103	0.0000000054
5	Toronto	Canada	toronto.ca	2010-01-01 (4 years)	-6	1;2;3	25	6;9	9.5	86.523	75.414	0.128	0.0000000000
6	Munich	German	muenchen.de	2010-01-01 (4 years)	-4	1;2;11	24	6;7;8	10.0	130.090	123.034	0.054	0.0118703200
7	Warsaw	Poland	um.warszawa.pl	2012-01-01 (2 years)	-4	1;2;11	25	5;6;8	10.5	18.503	17.349	0.062	0.0429774500
8	Berlin	German	berlin.de	2010-01-01 (4 years)	-2	1;2;11	23	6;7;8	10.5	360.514	345.562	0.041	0.0126842700
9	Copenhagen	Denmark	kk.dk	2010-01-01 (4 years)	-1	1;2;3	22	7;8	10.5	34.167	41.407	-0.212	0.0000000000
10	Hamburg	German	english.hamburg.de	2010-01-01 to 2012-12-	-1	1;2;3	23	6;7;8	11.0	164.044	160.226	0	insignificant
11	Dublin	Ireland	dublincity.ie	2010-01-01 (4 years)	2	1;2;10	20	7	11.0	20.762	18.663	0	insignificant
12	Chicago	USA	cityofchicago.org	2010-01-01 (4 years)	-6	1;2;3	29	5	11.5	35.758	29.181	0.184	8.398585e-08
13	Paris	France	paris.fr	2010-01-01 (4 years)	2	1;2;11	25	5;6;9	13.5	132.813	125.520	0.055	0.0319001400
14	New York	USA	council.nyc.gov	2010-01-01 (4 years)	-2	1;2;3	30	5;10	14.0	318.148	311.417	0	insignificant
15	London	UK	londoncouncils.gov.uk	2010-01-01 (4 years)	5	1;2;3	23	6;7;8;9	14.0	3.762398	4.163	-0.106	0.0032965660
16	Washington	USA	wa.gov	2010-01-01 (4 years)	-2	1;2;3	32	4;10	15.0	394.6311	420.0565	-0.064	5.29E-07
17	Istanbul	Turkey	ibb.gov.tr	2010-01-01 (4 years)	3	1;2;3	29	5;10	16.0	95.339	95.451	0	insignificant
18	Tokyo	Japan	metro.tokyo.jp	2010-01-01 (4 years)	2	1;3	31	5;10	16.5	606.386	626.752	0	insignificant
19	Los ángeles	USA	council.lacity.org	2010-01-01 (4 years)	9	2;3	25	6;7;10;11	17.0	28.324	35.411	-0.250	0.0000000000
20	Barcelona	Spain	bcn.cat	2010-01-01 (4 years)	5	1;2;3	29	5;10	17.0	73.000	81.075	-0.111	0.001385713
21	Madrid	Spain	madrid.es	2011-01-01 (3 years)	2	1;2;11	33	5;10	17.5	60.813	57.147	0.060	0.0079568270
22	Osaka	Japan	city.osaka.lg.jp	2010-01-01 (4 years)	3	1;3	33	4;10	18.0	46.785	46.610	0	insignificant

Table 7. A comparison of the traffic on city government sites between comfortable and cold months. The p-value is obtained using an ANOVA test. The Benjamini & Hochberg (3) procedure was followed to control the false discovery rate set at Q=0.20.

Section 10: Server failure, outliers and lost data

10.1 Server failure

Because the data collection in Section 2 is performed automatically by our script at midnight, serve failure took place occasionally. During 16 months of data collection (488 dates), due to server failure we did not get data for 13 dates (2.67%). The random server failure could happen at either our side or at the website serve side. Because daily revenue of the date N is calculated by obtaining the difference of sale of date N-1 and date N, 25 dates (5.1%) were dropped from analysis. Therefore, 463 days are retained for the final analysis.

The 13 missing dates from our dataset are: 2012-09-11, 2012-09-12, 2012-12-28, 2013-01-07, 2013-01-30, 2013-02-26, 2013-04-01, 2013-04-03, 2013-04-08, 2013-04-20, 2013-05-24, 2013-06-14, 2013-09-14. We use the mean-replacement method to obtain transaction values for the days of server failure when plotting daily revenue in Figure 4.

10.2 Outliers

The revenue of the site is found to usually peak at the end of each month frequently. Therefore, we remove 8 dates with daily revenue of over 25.000.000 RMB (2.9 Million Euro) for 16 months: 2013-02-25, 2013-04-27, 2013-04-26, 2013-06-28, 2013-08-27, 2013-08-28, 2013-08-29, 2013-10-29. As shown in Figure 4, there is a sharp drop of revenue during 2013/02/09 – 2013/02/11, due to Chinese spring festival. Therefore, these dates are removed from analysis as well. In total 11 dates are removed from the total 463 days. Therefore 452 days are retained after removing the outliers. Based on 452 days of 28 cities, we obtain 12656 city-date pairs.

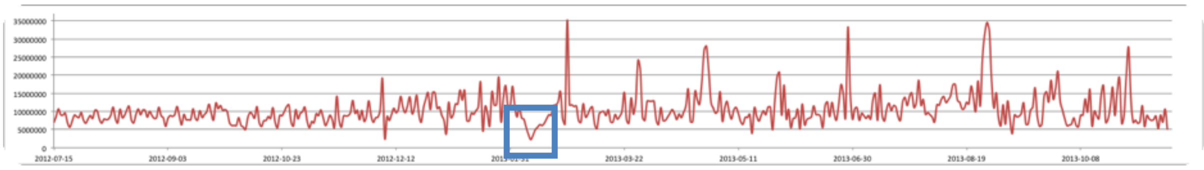

Figure 4. Transaction revenue of Lashou.com of 16 months (Blue rectangle annotates the sharp drop of revenue in Chinese spring festival).

10.3 Lost data

On occasion, the Lashou API failed to return data for particular cities for particular dates, most likely due to possible network problems or the limitation of the reading capability of receiving server. As a result, of the possible 12656 city-date pairs we obtained 12548 (99.15%). Therefore, on average, we obtained 448 days of data (std. = 2.76) for each city. In other words, on average, about 2 days of data were lost (days N+1 will be removed accordingly) due to connectivity issues for each city for 452 days.

SI References

1. Lawrence MG (2005) The relationship between relative humidity and the dewpoint temperature in moist air: a simple conversion and applications. *Bulletin of the American Meteorological Society* 86.

2. Kawamura T (1965) Distribution of discomfort index in Japan in summer season. *J Meteorol Res* 17:460-466.

3. Benjamini Y, and Hochberg Y (1995) Controlling the false discovery rate: a practical and powerful approach to multiple testing. *Journal of the Royal Statistical Society. Series B (Methodological)*:289-300.

4. Pfafferoot JU, Herkel S, Kalz D, and Zeuschner A (2007) Comparison of low-energy office buildings in summer using different thermal comfort criteria. *Energy and Buildings* 39:750-757.
